# Supercritical CO_2_ and Conventional Extraction of Bioactive Compounds from Different Cultivars of Blackberry (*Rubus fruticosus* L.) Pomace

**DOI:** 10.3390/plants13202931

**Published:** 2024-10-19

**Authors:** Indrė Čechovičienė, Kiril Kazancev, Ewellina Hallmann, Eglė Sendžikienė, Marcin Kruk, Jonas Viškelis, Živilė Tarasevičienė

**Affiliations:** 1 Department of Plant Biology and Food Sciences, Agriculture Academy Vytautas Magnus University, Donelaičio Str. 58, LT-44248 Kaunas, Lithuania; 2 Department of Environment and Ecology, Faculty of Forestry and Ecology, Agriculture Academy Vytautas Magnus University, Donelaičio Str. 58, LT-44248 Kaunas, Lithuania; 3 Department of Functional and Organic Food, Institute of Human Nutrition Sciences, Warsaw University of Life Sciences, Nowoursynowska Str. 159C, 02-776 Warsaw, Poland; 4 Bioeconomy Research Institute, Agriculture Academy, Vytautas Magnus University, Donelaičio Str. 52, LT-44248 Kaunas, Lithuania; 5 Department of Food Gastronomy and Food Hygiene, Institute of Human Nutrition Sciences, Warsaw University of Life Sciences, Nowoursynowska 159c, 02-776 Warsaw, Poland; 6 Lithuanian Research Centre for Agriculture and Forestry, Institute of Horticulture, Kaunas Str. 30, Kaunas District, LT-54333 Babtai, Lithuania

**Keywords:** blackberry pomace, extracts, fatty acids, carotenoids, chlorophylls, volatile compounds

## Abstract

The main objective of this work was to extract bioactive compounds from different cultivars of blackberry pomace using conventional Soxhlet and supercritical CO_2_ (SC-CO_2_) extraction methods. For Soxhlet extraction, two different solvents, ethanol and *n*-hexane, were used. Qualitative and quantitative composition of fatty acids was determined by GC, carotenoids and chlorophylls by HPLC, and volatile organic compounds were identified with an e-nose based on GC. The yield of the extract was influenced by the extraction, while the qualitative content of the extracts was also dependent on the cultivar. While there were no differences in the types of fatty acids extracted, their content varied significantly depending on the cultivar, extraction method, and their interaction. The results showed that linoleic acid (C18:2), oleic acid (C18:1), and α-linolenic acid (C18:3) were the most prevalent in all cultivars of blackberry pomace extracts. The linoleic acid content varied from 33.33 to 64.77% depending on the variety, and the ratio of omega-6 to omega-3 varied from 3.17% to 5.71%. Significantly higher quantities of carotenoids and chlorophylls were obtained in Soxhlet extraction with *n*-hexane in all extracts. The major carotenoid in the ‘Orkan’ and ‘Polar’ extracts was lutein, while in the ‘Brzezina’ extract, it was β-carotene. The extraction method has a significant impact on the flavor profile of the extracts.

## 1. Introduction

Food waste, one of the largest sources of waste in the world, is a very important origin of biologically active compounds that can be used in various products such as functional food, nutrition, and cosmetics [1,2,3]. Blackberry has a high content of various bioactive compounds that have antioxidant, anticancer, anti-inflammatory, and antineurodegenerative biological properties [3,4,5,6,7]. 

However, the food industry usually removes the seeds when processing blackberries because they contain many seeds that consumers do not like [8]. The processing of blackberries produces about 20–30% pomace, which consists of seeds, skin, and stems [1]. One of the possible products that can be obtained from blackberry pomace is its oil [9]. The valorization of pomace and its transformation into new products can contribute to mitigating environmental problems and to sustainable development [10]. In previous research, several ways of using pomace in food production have been identified. Pomace can be utilized to prolong the shelf life of ground meat [11]. This research is in line with other research, as reported by Diez-Sánchez et al. [12] and Campos-González et al. [13], regarding using pomace in meat products, and as another way to enrich low-value food products such as biscuits with biologically active compounds [14], and other researchers reported that pomace can be used in different bakery products [12,15,16,17]. According to Sharma et al. [18] and Bechtold et al. [19] pomace can be valorized to produce natural pigments. The production of lipophilic extracts could be another possible method for the valorization of blackberry pomace [20]. Among the value-added bioactive components obtained from blackberry lipophilic extracts are carotenoids, which are potential colorants that are synergistic with antioxidant capacity and can greatly contribute to the development of functional foods by replacing the synthetic pigments [20]. According to Kitryte et al. [21], blackberry seeds contain an oil that is mainly polyunsaturated fatty acids [21] and is rich in bioactive nutritional compounds of linoleic, linolenic fatty acids and has extremely high levels of stearidone fatty acid, which is a powerful fatty acid that increases eicosapentaenoic acid levels in tissues (20:5n-3) and docosahexaenoic acid (20:5n-3) [22].

The process of extracting and purifying phytochemicals and antioxidants from pomace is typically influenced by various factors, such as time, temperature, solvent concentration, and solvent polarity [23]. The researchers utilize a serial comprehensive extraction method, which involves sequentially extracting compounds using solvents with increasing polarity, starting from a non-polar (*n*-hexane, CO_2_) and progressing to a more polar solvent (water or ethanol). This approach ensures the extraction of a broad range of compounds with varying polarities, as no single solvent is effective in extracting all phytochemicals and antioxidant compounds [23]. Ethanol as an organic solvent for the extraction of polyphenols from fruit by-products is widely used, but *n*-hexane for oil extraction is hazardous to workers and the environment, and alternative extraction procedures and solvents may be required [24]. Supercritical carbon dioxide has been widely proposed as a solvent for oil extraction [9,25]. SC-CO_2_ treatment is becoming increasingly relevant for large-scale extraction for the production of various types (lipids and other bioactive substances) used in food, pharmaceutical, cosmetic, and other high-value fields [26]. Carbon dioxide is a safe solvent because it is non-flammable, non-toxic, and affordable, and it also allows for supercritical operation at low pressures and temperatures. The materials extracted with SC-CO_2_ are mostly non-polar, such as oils extracted from seeds [27]. For the extraction of fatty acids, the most commonly used methods are Soxhlet with organic solvents (such as ethanol or *n*-hexane) and SC-CO_2_. The supercritical carbon dioxide extraction method is used because, due to the low oxidative and thermal effects, it produces high quality oils that often do not require further refining, and it preserves the original properties and prevents contamination by residual liquid solvents [26,28]. According to Campalani et al. [26], supercritical carbon dioxide extraction is more selective in obtaining high-quality fatty acids than the conventional Soxhlet method using *n*-hexane as solvent. A greater extraction yield does not always indicate that the oils obtained are of high quality. The oil obtained by *n*-hexane extraction is treated with refining processes such as solvent removal [9]. The Soxhlet method has many advantages for people such as safety, profitability, and a lower toxicity for the investigator and the environment, but there are disadvantages: it uses a long extraction time, risks possible degradation of polyphenols [29], and the extract may have unpleasant aromas or color [30]. Other researchers use supercritical fluid extraction (SFE) with carbon dioxide as a solvent to extract oil from blackberry; it is an environmentally friendly technology that offers important advantages over conventional extraction methods [31]. According to Perdomo et al. [31], the SFE method with carbon dioxide as a solvent yielded very pure blackberry oil extracts with interesting biologically active prospects for its use in the food, pharmacological, or cosmetic industries. Campalani et al. [26] report that oils extracted from blackberry seeds using SC-CO_2_ extraction were purer and richer in essential fatty acids (26% selectivity towards fatty acids) than those extracted by the conventional method with *n*-hexane (1.4% selectivity towards fatty acids). According to Ispiryan et al. [32], SC-CO_2_ extraction might preserve the structural components (fiber content) of the seeds after extraction greater than other extractions. Extraction of raspberry seeds using the SC-CO_2_ extraction method shows the highest protein content of oil and nitrogen, which are major components of amino acids in proteins, and this method also reduces the fat content of the seeds the most after extraction, compared to other methods (cold pressing, extraction with *n*-hexane and subcritical CO_2_) [32]. Other researchers [33] found that the highest yield of oil from raspberry (*Rubus idaeus* L.) seeds was obtained by the SC-CO_2_ extraction method—18.81%; this oil was characterized by a high content of fatty acids, especially α-linolenic (ω-3) and palmitic acid—but in oil obtained by solvent (*n*-hexane), extraction had the highest amount of linoleic (ω-6)—44.8 mg 100 g^−1^—and oleic acid(ω-9)—10.6 mg 100 g^−1^. However, the SC-CO_2_ extracted oil released the highest amount of total carotenoids (3.25 mg 100 g^−1^) and different tocopherols such as γ-tocopherol (26.4 mg 100 g^−1^), α-tocopherol (3.2 mg 100 g^−1^), and amount of δ-tocopherol (1.8 mg 100 g^−1^) [33]. According to Gil-Martínez, L. et al. [34] blackberry ethanolic extracts (conventional method) confirmed their potential use in the prevention of oxidative stress and inflammation; moreover, ethanolic blackberry extract can be a source of antimicrobial agents, molecules that can enhance the immune system’s ability to combat infections, and may also be used for the prevention and treatment of various colorectal tumors. 

Researchers’ studies provide information on the advantages and disadvantages of different extraction methods (conventional, SC-CO_2_, etc.), and analyze the technological parameters of the bioactive compounds from seeds or waste extraction process. However, most of the studies analyzing seed oils focus on fatty acid, sterol, and tocopherol analyses, while there is lack of study describing the carotenoids, chlorophylls and volatile compounds from blackberry pomace oils. The primary reason for the limited information on the thorough characterization of seed oils is the complex of various compound families, which have a broad range of polarities, making extraction and analyses difficult. Valorization must also be sustainable, economically viable, environmentally and human friendly, with a broader perspective on the use of the product obtained, which requires cultivar studies and qualitative analyses of the extracts, not just technological ones. 

Therefore, the aim of this work was to identify how different extraction methods (traditional, more environmentally and human friendly, etc.) for the extraction of lipophilic compounds from blackberry waste affect the composition of the extracts, assessing not only the efficiency of the extraction, but also the influence of cultivar on the composition of the extracts. Only one of the technological parameters was chosen for all the extraction methods, because the main objective was not to identify the best extraction conditions (to optimize them), as these methods have been studied, but rather to evaluate the qualitative parameters of the extracts and the less studied lipophilic compounds when the optimal ones have been chosen, including the influence of the cultivar.

## 2. Results

### 2.1. Extraction Yield of Blackberry Pomace

The findings revealed that the pomace oil extraction yield was significantly higher when the extraction was performed in a Soxhlet apparatus with ethanol, in the pomace of all blackberry cultivars (Table 1). Wajs-Bonikowska et al. [7] also determined that the highest extract yield was obtained with Soxhlet extraction using ethanol as a solvent (14.2%). According to McNichol et al. [35], a polar solvent (ethanol) resulted in higher lipid content (>25%), while the non-polar solvent *n*-hexane resulted in a lower yield (~3%), where the approximate yield ratio was 8.33. The extract yield ratio obtained in the research shows similar results, where the ratio of extract yield obtained in the Soxhlet apparatus using ethanol and *n*-hexane varied from 7.52 to 8.65 depending on the blackberry cultivar (Table 1).

According to Bada et al. [22], the extraction yield from blackberries in a Soxhlet apparatus using *n*-hexane as a solvent was 15.68%. In comparison, our Soxhlet with *n*-hexane and SC-CO_2_ extraction results in a slightly higher value determined in the Soxhlet with *n*-hexane extraction, but no statistically significant differences were found between these values (Table 1). According to Campalani et al. [26], higher extraction yield was found in Soxhlet using *n*-hexane as a solvent than with supercritical fluid extraction using CO_2_ as a solvent; the author claims that this is for two reasons: the longer extraction time with *n*-hexane compared to SC-CO_2_ and the higher *n*-hexane polarity as a solvent compared to CO_2_. According to Li et al. [36], extraction yield using ethanol was nearly three times higher than when using hexane as solvent. This only confirms that the polarity of the solvent results in a different composition of the extracts in relation to the compounds extracted. Ethanol-like solvent is usually used for the extraction of hydrophilic compounds due to its properties which allow it to dissolve polyphenols [37], and it is clearly seen that the Soxhlet extraction using ethanol extracted more than the lipophilic compounds, because the color of the extract is red, while the other two extracts match blackberry oil color—green/yellow (the image in Section 3.3.1). According to Krist et al. [38], blackberry seed oil is dark green to yellow because it contains chlorophylls. Blackberry cultivar had no significant effect on the extract yield. 

### 2.2. Peroxide Value of Blackberry Pomace Extracts

The peroxide value can be used to determine the level of decomposition of oils and fats [39]. The amount of peroxides shows the degree of oxidation of oil and fat; this value should not exceed 10 mEq kg^−1^ in fresh oil, but for some oils, it can be up to 20 mEq kg^−1^ [32]. Significant differences in the peroxide value of the extracts were obtained between different extraction methods, but not between cultivars (Table 2). The highest values of peroxides are determined in the Soxhlet extracts with *n*-hexane as a solvent, ranging from 3.39 in the pomace extract of the ‘Brzezina’ cultivar to 3.80 in the pomace extract of the ‘Polar’ cultivar. The lowest values are determined in the Soxhlet extracts with ethanol as a solvent, ranging from 1.53 in the pomace extract of the ‘Brzezina’ cultivar to 1.76 in the pomace extract of the ‘Polar’ cultivar. According to other researchers, the peroxide values in blackberry extracts can vary from 1.86 to 5.57 and from 4.44 to 5.8 mEqkg^−1^ [40,41]. According to Ispiryan et al. [32] the value of peroxide may vary by oil extraction method and technological parameters (temperature).

### 2.3. Antioxidant Activity, Total Phenolic Content (TPC), Total Flavonoid Content (TFC) and Total Anthocyanin Content (TAC) 

Most of the phenolic compounds are hydrophilic and are not present in large amounts in berry seed oils [42], for this reason, there is a lack of sources describing the amounts of polyphenolic compounds (TPC, TFC, TAC, and antioxidant activity) in blackberry pomace lipophilic extracts.

Phenolic content in Soxhlet ethanol extract was dependent on cultivar. The total phenolic content in the Soxhlet ethanol extract varied from 313.04 mg 100 g^−1^ in the pomace extract of ‘Polar’ cultivar to 347.17 mg 100 g^−1^ in the pomace extract of the ‘Brzezina’ cultivar (Table 3). The TFC in our results varied from 133.75 mg 100 g^−1^ in the ‘Polar’ cultivar to 158.89 mg 100 g^−1^ in the extract of the ‘Brzezina’ cultivar pomace.

Anthocyanins are the largest group of flavonoids in red-, blue-, and purple-colored berries [43], but they are also very sensitive bioactive compounds, and their effectiveness can be determined by different factors such as temperature, pH, and the presence of other related compounds [44]. In our results, the TAC varied from 26.33 to 97.06 mg 100 g^−1^ for pomace extracts (Table 3). During the research, it was determined that the extract from the ‘Polar’ cultivar pomace was slightly browner than red, which may have contributed to the lowest anthocyanin content being in the pomace extract of this cultivar. 

Earlier research [45] shows that the pomace of the ‘Polar’ cultivar had the lowest organic acid content (170.96 mg g^−1^); this may result in lower anthocyanin content in the extract of 26.33 mg 100 g^−1^, while in the ‘Orkan’ cultivar the highest organic acid content was found (273.83 mg g^−1^), as well as anthocyanin content, with 97.06 mg 100 g^−1^. According to the Oancea [46], organic acids generally increase the heat stability of anthocyanins during extraction and anthocyanins’ thermal stability may vary based on the presence of other compounds in the extracts such as sugars, salts, and other phenols. The differences in the total anthocyanin content in all cultivars’ blackberry pomace are statistically significant. 

The antioxidant activity of blackberry Soxhlet extracts with ethanol was measured as IC_50_; this index is inversely related to the antioxidant activity (Table 3). The extract of the ‘Brzezina’ cultivar pomace had the highest DPPH^•^ radical-scavenging activity (IC_50_ = 105.74 µg mL^−1^, *p* < 0.05) (Table 3). The IC_50_ index of antioxidant activity varied significantly (*p* < 0.05) between the cultivars.

### 2.4. Fatty Acid Profiles of the Extracts

Blackberry pomace contains a large amount of seed content (from 38.50 to 48.97%) and, as can be seen in a result, they are a source of lipophilic compounds. Blackberry seeds contain an average of 14–15% oil, resulting in a total lipid content of approximately 0.5 g 100 g^–1^ in the whole blackberry fruit [47]. No differences were found in the types of fatty acids, but their content varied considerably according to the cultivar and extraction method. The percentage of fatty acids in blackberry pomace extracts was very similar for all cultivars in n-hexane Soxhlet and SC-CO_2_ extracts. 

ANOVA analysis showed a significant (*p* < 0.05) influence among the fatty acids depending on the cultivar, as well as extraction method, and the cultivar and extraction method’s combined effect. The cultivar of the blackberry pomace affects the content of C4:0 (butyric acid), C10:0 (capric acid), C15:1 cis10 (pentadecenoic acid methyl ester), C18:2 (linoleic acid), C18:3 (α-linolenic acid), C21:0 (henicosanoic acid), C20:4 (eicosatetraenoic acid), and C20:3 11,14,17 (mead acid) fatty acids in the extracts. Cultivar differences were evident in the content of C18:2 fatty acid in the Soxhlet extract with ethanol (the highest content was observed in the ‘Polar’ cultivar and the lowest in the ‘Brzezina’ cultivar extract). The lowest C18:3 fatty acid content was found in the extracts of the ‘Polar’ cultivar, while the highest C21:0 content was found in the ‘Orkan’ Soxhlet with ethanol extract (Table S1).

Considering the influence of extraction on the fatty acid profile, several significant differences in amounts were observed. The Soxhlet extract with ethanol differs the most from the other two extracts (Soxhlet with n-hexane and SC-CO_2_) in terms of fatty acid composition, containing the maximum amounts of the following fatty acids in all cultivars’ extracts: C6, C11, C12,C14, C14:1 cis-9, C15, C16, C16:1 cis-9, C17, C17:1 cis-10, C20:3 8,11,14, C20:3 11,14,17, C20:5, C23, C24:1, and C22:6 (Table S1). The extraction method does not affect the content of C17:0 (margaric acid), C18:0 (stearic acid), C18:1 (oleic acid), C18:3 (α-linolenic acid), C20:1 (eicosenoic acid), or C20:2 (eicosadienoic acid) fatty acids, but the combination of cultivar and extraction method affects the content of C8:0 (caprylic acid), C10:0 (capric acid), C13:0 (tridecylic acid), C14:1 cis-9 (physeteric acid), C15:1 cis-10 (pentadecenoic acid methyl ester), C18:2 (linoleic acid), C19:0 (nonadecylic acid), C21:0 (henicosanoic acid), and C20:3 11,14,17 (icosatrienoic acid) fatty acids (Table 4). Both factors and interactions of factors influenced the content of fatty acids C10:0 (capric acid), C15:1 cis10 (pentadecenoic acid methyl ester), C18:2 (linoleic acid), C21:0 (henicosanoic acid), and C20:3 11,14,17 (mead acid). Whereas fatty acids C18:0 (stearic acid), C18:1 (oleic acid), C20:1 (eicosenoic acid), and C20:2 (eicosadienoic acid) were not affected by any single factor or their interactions.

Regarding all the results, it was found that linoleic acid (C18:2) is the most abundant in blackberry pomace extracts of all cultivars; its amount varies from 33.33% (‘Brzezina’ Soxhlet with ethanol extract) to 64.77% (‘Orkan’ Soxhlet with n-hexane extract). Bada et al. [22] also determined that linoleic acid is the most notable finding in blackberries (67.96 ± 1.96 g 100 g^−1^ oil). 

Significant amounts of C18:1 (oleic acid) were also identified, ranging from 10.41% (‘Orkan’ Soxhlet with n-hexane extract) to 22.24% (‘Brzezina’ Soxhlet with ethanol extract), but no statistical differences were found between different extraction methods and cultivars. Blejan et al. [10] determined that blackberry pomace contains 17.79% oleic acid.

According to Wajs-Bonikowska et al. [7], blackberry seed oil has been shown to be of a high quality due to its high content of polyunsaturated fatty acids such as omega-6 (linoleic acid) and omega-3 (linolenic acid), which are 42–64% and 14–18%, respectively. The C18:3 (α-linolenic acid) was also found among the predominant fatty acids and it ranged from 9.67% (‘Brzezina’ Soxhlet with ethanol extract) to 13.22% (‘Orkan’ Soxhlet with n-hexane extract). Van Hoed et al. [48] also reported similar results for α-linolenic acid, with 17.53% in blackberry seed oil. The amount and composition of oil in blackberry pomace depends on the climate and geographical location where blackberries were grown [22]. According to our results, the composition is also highly dependent on the cultivar, extraction method, and the solvent chosen (Table S1). According to Wei et al. [49], higher levels of saturation in oils extracted by the Soxhlet method could be caused by the oxidation of unsaturated lipids initiated by the extraction temperature and time. 

When the results of the individual fatty acid groups (SFA—saturated fatty acids, MUFA—monounsaturated fatty acids, PUFA—polyunsaturated fatty acids) are summarized, the same predominant acids can be seen between all extraction methods and cultivars. The predominant acid of the SFA group is C16:0 (palmitic acid), with a range of 3.66–7.46%; the MUFA group is C18:1 (oleic acid), ranged 10.41–22.24%; and the predominant acid of PUFA group is C18:2 (linoleic acid), ranged 33.33–64.99% (Table S1). 

Soxhlet with ethanol extracts have a higher content of SFA and lower content of PUFA in all cultivar pomace extracts (Table 4). According to Li et al. [36], Soxhlet extraction of lipids showed a significant difference in extraction efficiency between hexane and ethanol; because ethanol is a polar solvent, it can extract more polar lipids such as SFA [36], and other bioactive compounds, such as oil-soluble vitamins, phytosterols, tocopherols, and pigments [50]. Soxhlet with ethanol produces extracts rich in polar lipids, whereas hexane extracts are more commonly rich in neutral lipids (triacylglycerols) [50]. According to McNichol et al. [35], ethanol showed a higher variability in lipid content compared to the other solvents (acetone, hexane).

The cultivar and the interaction of the cultivar and extraction method influenced the content of MUFAs and PUFAs, except SFAs, while the extraction method affected the content of all three fatty acids group (SFA, MUFA, and PUFA) (Table 4).

The content of omega-3 acids was affected by cultivar and the interaction of cultivars and extraction methods, but were not affected by extraction method, while omega-6 acids and omega-9 acids were affected by all factors and their interactions (Table 4). According to Fang et al. [51], blackberry seed oil is an excellent source of linoleic acid and essential fatty acids. According to De Filette et al. [52], blackberry seed oil is rich in PUFA.

Extracts of all blackberry cultivars’ pomaces have an overall high level of PUFAs, which provide essential fatty acids (EFAs) [48] and are rich in MUFAs. Essential fatty acids cannot be synthesized in the body through any known chemical process, so they must be acquired through the diet. The ratio of n-6 to n-3 fatty acids (n-6/n-3) has been linked to cancer, heart disease, cardiovascular disease, hypertension, autoimmune disease, arthritis and other inflammatory diseases [48,53]. The ratio of n-6 to n-3 is recommended to be lower than 10 [50,54], but, in Western countries, a ratio of 15:1 is recommended. According to Demler et al. [54], the recommended ratio is 5:1, whereas according to the Simopoulos et al. [55], the recommended omega-6/omega-3 ratio for health benefits stands between 1 and 5, and others authors recommend a ratio of 4:1 [48,56]. A high content of a-linolenic acid (>50%) leads to autoxidation and degradation, and also to unpleasant odors. During the degradation process, the first odorless monohydroperoxides are formed from PUFA [53]. According to Radojač et al. [40], blackberry seed oil contains MUFA from 17.87 to 19.97% and PUFA from 74.94 to 78.56%; these results are similar to ours, where MUFA varied from 11.25% (‘Orkan’ Soxhlet with *n*-hexane) to 26.76% (‘Brzezina’ Soxhlet with ethanol) and PUFA varied from 47.46% (‘Brzezina’ Soxhlet with ethanol) to 78.55% (‘Orkan’ SC-CO_2_ extraction). Nearly all samples exhibited a high proportion (over 60%) of polyunsaturated fatty acids, a distinctive feature of blackberry extract. Our SFA results are higher (from 10.01% for ‘Brzezina’ Soxhlet with *n*-hexane to 25.78% for ‘Brzezina’ Soxhlet with ethanol) than those published by Radojač et al. [40]—from 7.13% to 7.53% in blackberry seed oil from differently treated seeds (temperature and time). The ratio of omega-6 to omega-3 varied from 3.17% to 5.71% and these results are similar to those reported by Radojač et al. [40], where the ratio of these fatty acids varied from 4.25% to 4.45%. Low values of n-6/n-3 indicate the possible use as food ingredients due to a decrease in the ratio of n-6/n-3 fatty acids [57]. Wajs-Bonikowska et al. [7], after the extraction of blackberry pomace, reported that SFA content in SC-CO_2_ extract was 7.2%, Soxhlet *n*-hexane was 9.0%, and Soxhlet with ethanol extraction was 5.8%; for MUFA: SC-CO_2_ extract was 12.6%, Soxhlet *n*-hexane was 14.1%, and ethanol extraction was 7.6%; PUFA: SC-CO_2_ extract was 58.2%, Soxhlet *n*-hexane was 57.7%, and ethanol extraction was 30.4%; and for the ratio of omega-6 and omega-3: SC-CO_2_ extract was 4.5%, Soxhlet *n*-hexane was 2.7%, and ethanol extraction was 2.7%. According to Matei et al. [58], blackberry oil extracted with *n*-hexane had 7.34% SFA, 16.52% MUFA, 76.14% PUFA, and n-6/n-3 was 5.73%. According to Li et al. [59], a favorable ratio of n-6/n-3 fatty acids in blackberry seed oil was 1.49–3.86; these results are similar to our 3.17–5.92. Our results show that the lowest height ratio of MUFA/PUFA was in the Soxhlet extract with ethanol in all cultivars’ pomaces, while the ratios of PUFA/SFA, n-6/n-3, and U/S was highest in the two other extracts. 

ANOVA analysis showed a significant (*p* < 0.05) influence among fatty acid composition (SFA, MUFA, PUFA, omega-3, omega-6, and omega-9) that equally dependent on cultivar, extraction method, and their combined effect.

### 2.5. Corotenoids and Chlorophylls in Blackberry Pomace Extracts

Carotenoids are recognized for their important role in human health and disease prevention; dietary intake of lutein and zeaxanthin reduces the risk of cancer and cardiovascular disease [60]. In plants, carotenoids perform such important functions as photosynthesis, photoprotection, hormone precursors, and aroma and flavor production [61]. They are described as lipid-soluble pigments [62]. Plant-based colorants such as β-carotene, lutein, other carotenoids, and chlorophylls can be used to impart different colors to foods [63]. Natural carotenoid pigments such as lutein, zeaxanthin, α-carotene, and β-carotene impart colors ranging from yellow to red [64]. ANOVA analysis showed a significant (*p* < 0.05) influence between carotenoids and chlorophylls, which was dependent on cultivar, extraction method and their combined effect.

Significantly higher recoveries of carotenoids and chlorophylls were obtained in extracts of pomace from all blackberry cultivars in Soxhlet with *n*-hexane extraction than in Soxhlet with ethanol (results for carotenoids and chlorophylls are 13.2 and 9.23 times lower, respectively) and SC-CO_2_ extraction (results for carotenoids and chlorophylls are 2.6 and 1.4 times lower, respectively). The highest content of total carotenoids (831.58 mg 100 g^−1^) and total chlorophylls (15.44 mg 100 g^−1^) was found in the Soxhlet with *n*-hexane extract of the ‘Orkan’ cultivar, while the lowest amounts of total carotenoids (33.54 mg 100 g^−1^) and total chlorophylls (1.35 mg 100 g^−1^) were found in the Soxhlet extracts with ethanol in ‘Polar’ and ‘Brzezina’ cultivars extracts, respectively. The main carotenoid in the extracts of the ‘Orkan’ and ‘Polar’ cultivar was lutein, while in ‘Brzezina’ it was β-carotene. When comparing different extraction methods, β-carotene and chlorophyll *b* were the major compounds in the Soxhlet with ethanol and SC-CO_2_ extracts (Table 5). According to Albuquerque et al. [62], the main carotenoid found in fruits and berries is β-carotene. According to Correa et al. [9], in blackberry seed oil, β-carotene ranged from 8.50 to 44.77 mg 100 g^−1^. In our research values are much higher and ranged from 20.51 mg 100 g^−1^ (‘Polar’ Soxhlet with ethanol extract) to 170.83 mg 100 g^−1^ (‘Orkan’ Soxhlet with *n*-hexane extract). Soxhlet with *n*-hexane extracts showed a higher efficiency in extracting β-carotene in the ‘Orkan’ and ‘Polar’ cultivars pomace extracts.

Chlorophyll is a fat-soluble compound and is mainly extracted in a non-polar or organic solvent [63]. Chlorophyll *a* and *b* are most abundant in various plant tissues and in fruits [62]. Chlorophyll *a* produces a blue–green color and chlorophyll *b* produces a yellow–green color [62]. Numerous scientific studies describe chlorophylls in blackberry leaves [65,66,67,68], but there is a lack of information sources on chlorophylls in blackberry pomace and/or oils. Our research results show that the highest contents of chlorophyll *a* (5.78 to 8.37 mg 100 g^−1^) and chlorophyll *b* (5.85 to 7.45 mg 100 g^−1^) were found in extracts obtained by Soxhlet with *n*-hexane, and the lowest results were found in the extracts obtained in Soxhlet with ethanol; as such, the color of these extracts was red (the image in Section 3.3.1) because that ethanol separated not only the lipophilic but also the hydrophilic fraction from blackberry pomace [37] (Table 3). Blackberry seed oil typically ranges from green to yellow in color due to the presence of chlorophylls [38]. The stability and color of chlorophyll pigments depend on enzyme activity and acidity [69].

### 2.6. Volatile Organic Compounds (VOCs)

**Figure 1:**
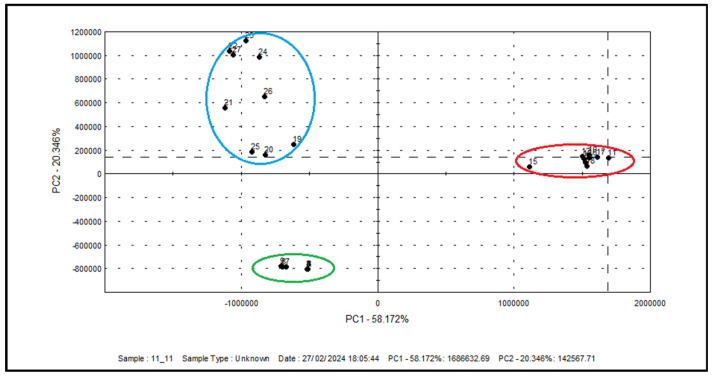
Principal component analysis (PCA) for organic volatile compounds in different blackberry extracts. Marked in blue color are Soxhlet extracts with ethanol; marked in red color are Soxhlet extracts with *n*-hexane; marked in green color are SC-CO_2_ extracts.

Differences in the volatile composition of samples of blackberry extracts by different extraction methods were observed (Figure 1). All extracts are completely separated into three groups according to the used solvent. All blackberry pomace extracts made with Soxhlet with *n*-hexane were situated at positive PC1 values (red color), while Soxhlet with ethanol samples were at positive PC2 (blue color), and SC-CO_2_ extracts were at negative PC1 (green color). These results show that the flavor characteristics of the extracts were significantly influenced by the extraction method. Two latent variables are included, accounting for 58.172% of the variance in the X matrix and explaining 20.346% of the variance in the Y matrix.

## 3. Materials and Methods

### 3.1. Materials

Blackberries of the cultivars ‘Polar’, ‘Orkan’, and ‘Brzezina’ were obtained from a farmer in the Joniškis region (56.30219045284591, 23.603429519328024) in Lithuania. The juice was extracted using a Stollar Commercial juicer (Riga, Latvia), and the pomace was freeze-dried in a lyophilizer ZIRBUS (Bad Grund (Harz), Germany) at −55 °C for 48 h. Following lyophilization, the freeze-dried blackberry pomace was ground with a food mill (Model Retsch ZM200, Haan, Germany) to a particle size of 0.2 mm and stored in sealed bags at −38 °C degrees until the analyses. Dry matter content of blackberry pomace: Polar—97.92, Orkan—97.80, and Brzezina—97.14%.

### 3.2. Solvents and Chemicals

The ethanol of an agricultural origin used for extraction was obtained from the MV group (Kaunas, Lithuania). The reagents listed below were utilized in this study: acetonitrile, acetone, carotenoid standards (beta-carotene, lutein, zeaxanthin, alpha-carotene, beta-cryptoxanthin), chlorophyll standards (chlorophyll a, chlorophyll b), magnesium carbonate, methanol, t-butyl methyl ether, trimethylsulfonium hydroxide, *n*-hexane ((Sigma-Aldrich Company (Poznan, Poland)), deionized water ((Milli-Q system (Millipore)), ethyl acetate ((Merck (Poznan, Poland)), Gallic acid (97%), Folin–Ciocalteu reagent, (3,4,5-trihydroxybenzoic acid, 99%), 2,2-diphenyl-1-picrylhydrazyl hydrate free radical (DPPH•, 95%), 6-hydroxy-2,5,7,8-tetramethylchroman-2-carboxylic acid (Trolox, ≥97%), aluminum chloride, and acetic acid from ((Sigma–Aldrich (Steinheim, Germany)). Sodium carbonate, potassium chloride, and sodium acetate were purchased from Enola. CO_2_ gas was also purchased ((BIOGON^®^ C Linde-gas (Vilnius, Lithuania)). All solvents were of analytical and HPLC grade.

### 3.3. Oil Extraction 

#### 3.3.1. Soxhlet Extraction 

**Figure 2:**
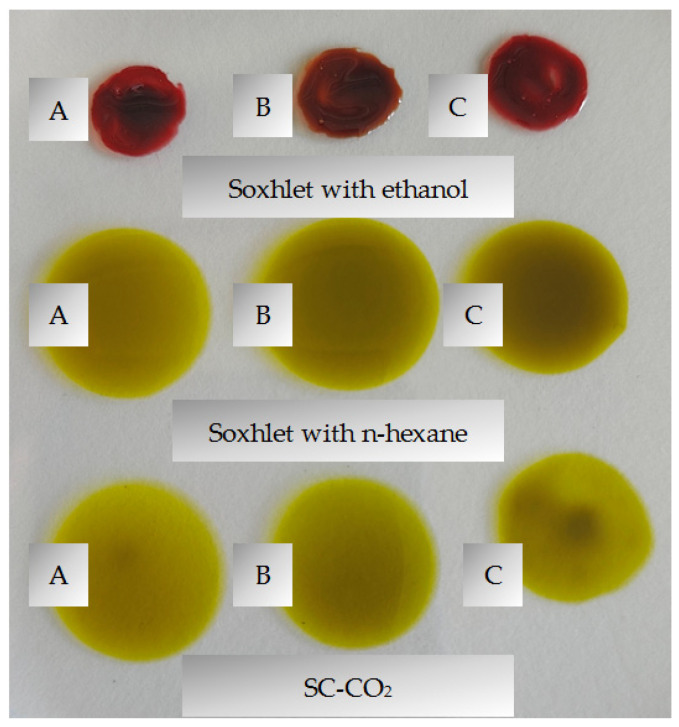
Blackberry pomace extracts (A—‘Orkan’, B—‘Polar’, C—‘Brzezina’ cultivars).

Cellulose thimbles were filled with 50 g of dry-ground pomace and 250 mL of solvent (*n*-hexane and 96% ethanol), the mixture was subjected to extraction for 8 h. At the end of the extraction period, the extract was collected and filtered on Whatman paper (retention 8–12 µm) to remove hard particles from the pomace, then the organic solvent was evaporated in a rotary evaporator at 55 °C (ethanol 240 min., *n*-hexane 150 min). The extracts (Figure 2) were stored in a freezer at −28 °C in black glass bottles until analysis.

#### 3.3.2. Supercritical Fluid Extraction (SC-CO_2_)

The supercritical fluid extraction was carried out using the SFT-150 supercritical fluid extractor (Supercritical Fluid Technologies, Newark, DE, USA). Each sample of 50 g was placed in a 150 mL thick-walled cylindrical stainless steel extractor vessel with 5-micron frits. To avoid the clogging of the system, the sample was placed between two layers of cotton wool. The vessel was heated with band heaters. The temperature of the extraction vessel was 40 ± 2 °C, with a pressure of 40 ± 2 MPa. A gas flow meter Gallus 2000 (Schlumberger Industries, Paris, France) was used to measure the volume of CO_2_ consumed and was expressed in standard liters per minute (SL/min). The flow rate was 1.2 SL/min. The process involved static extraction for 2 h and dynamic extraction for 6 h. The static extraction time was included in the total extraction time of 8 h. Extracts were stored in a freezer at −28 °C in black glass bottles until analysis [70].

### 3.4. Extraction Yield

The yields of the extracts were calculated according to Xiao et al. [71] as follows:

$$Extraction\;yield\;\left(\%\right)=\left(\frac{the\;final\;extract\;g}{\begin{array}{c} \;the\;dried\;pomace\;powder\;\\ g \end{array}}\right)\times 100$$

### 3.5. Oxidative Stability and Antioxidant Activity 

#### 3.5.1. Peroxide Value (PV)

The peroxide value was assessed using the standard method and expressed as meq O_2_/kg of extract [72].

#### 3.5.2. DPPH• Radical Scavenging Activity (DPPH^•^-RSA) of Ethanolic Soxhlet Extract

The antioxidant capacity of the DPPH^•^ assay was determined according to Brand-Williams et al. [73], with some adjustments. A Soxhlet ethanolic extract of pomace was dissolved in 10 mL of an ethanol/water mixture (50:50 *v*/*v*) and was prepared in 5 concentrations (from 50 to 250 µg mL^−1^). A 5 mL solution of DPPH^•^ radicals in ethanol with an absorbance of 0.800 ± 0.002 was added to 1 mL of blackberry pomace extract. The solution mixture was allowed to stand in the dark for 30 min. The absorbance of the solutions was measured using a Spectro UVD-3200 (Spectro UV-VIS Double Beam PC, Labomed, Los Angeles, CA, USA) spectrophotometer at a wavelength of 517 nm. As a blank, ethanol (96%) was used. The IC_50_ value indicates the concentration of sample needed to scavenge 50% of the DPPH^•^ radicals. Lower IC_50_ values suggest a greater ability to scavenge radicals [74].

### 3.6. Chemical Content of the Extracts

#### 3.6.1. Fatty Acid Profile of the Extracts

Analysis of fatty acid composition was executed according to Kazancev et al. [75] with some adjustments. Briefly, 500 μL of t-butyl methyl ether and 250 μL of trimethylsulfonium hydroxide (trimethylsulfonium hydroxide (TMSH)) were added to 10 mg ± 2 mg of extracted oil from blackberry pomace. 1 μL of the resulting solution was injected into a Perkin Elmer Clarus 500 gas chromatograph (GC/FID) (USA) using a split/splitless injector. An Alltech AT-FAME capillary column (30 × 0.25 mm × 0.25 μm) was connected to the chromatograph. Hydrogen was a gas carrier, pressure was constant at 90 kPa, and the split was set at 1:100.

#### 3.6.2. Carotenoid and Chlorophyll Content of the Extracts

Chlorophylls and carotenoids in the lipophilic extracts were measured using the HPLC method [76]. Briefly, 50 mL of extracts were mixed with cold acetone, and then magnesium carbonate was added. A cold ultrasonic bath (0 °C, 15 min) was used for the samples’ incubation. Following extraction, the samples were subjected to centrifugation (5500 rpm, 2 °C, 10 min). Briefly, 1 mL of the centrifuged extract was used for the analysis. The first mobile phase (A) contained 90% acetonitrile and 10% methanol, and the second phase (B) contained 68% methanol and 32% ethyl acetate following at a rate of 1 mL min^−1^. A time program was set up, of 1.00–14.99 min. phase A 100%; 15.00–22.99 min. phase A 40% and phase B 60%; and 24.00–32.00 min. phase A 100%. Detection was performed at 450 nm. Carotenoids and chlorophylls were identified by reference to external standards and expressed as mg 100 g^−1^ of blackberry pomace extract. 

#### 3.6.3. Total Phenolic Content (TPC) of Ethanolic Soxhlet Extract

The total phenolic content of the blackberry extract obtained by Soxhlet with ethanol was determined according to method described by Durdun et al. [77] with some modifications. Briefly, 0.2 g of extract was mixed with 10 mL of water/ethanol (30:70) solution, homogenized, and was allowed to stand in the dark for 24 h., then was filtered through Whatman paper (retention 8–12 µm). The Folin–Ciocalteu spectrophotometric method was used for the determination of the total phenol content of the extracts at 765 nm using a gallic acid as a calibration curve, and the results are expressed as mg 100 g^−1^ of blackberry pomace extract.

#### 3.6.4. Total Flavonoid Content (TFC) of Ethanolic Soxhlet Extract

The aluminum chloride colorimetric method was used to determine the total flavonoid content in blackberry extracts prepared by Soxhlet using ethanol as solvent. Briefly, 0.2 g of extract was mixed with 10 mL of water/ethanol (25:75) solution in an automatic shaker (Heidolph Vibramax 100, 31 W, Retsch GmbH, Haan, Germany) (1200 rpm) for 1 h, filtered using Whatman paper (retention 8–12 µm). Blackberry extract solution was mixed with 10 mL of aluminum chloride solution (2% *m*/*v*), 2 mL of ethanol (96%), and 1 mL of 1 M sodium acetate and incubated in the dark for 40 min. A Spectro UVD-3200 spectrophotometer (Spectro UV-VIS Double Beam PC, Labomed, Los Angeles, CA, USA) was used to measure the absorbance at a wavelength of 420 nm. Results are expressed as mg quercetin 100 g^−1^ of blackberry pomace extract [6].

#### 3.6.5. Total Anthocyanin Content (TAC) of Ethanolic Soxhlet Extract

The total anthocyanin content was determined by the pH differential method [78]. Briefly, 0.2 g of extracts prepared by Soxhlet using ethanol as solvent was added to 10 mL of ethanol (70%) and HCl (0.5%) in a ratio of 85:15. The solution mixture was allowed to stand in the dark for 24 h, and was then filtered through Whatman paper (retention 8–12 μm). The solution was added to 0.025 M potassium chloride solution (pH1) and 0.4 M sodium–acetate solution (pH4). Standard cyanidin-3-glucoside (449.2 g/mol) was used. Absorbances were then measured at wavelengths of 520 nm and 700 nm using a Spectro UVD-3200 spectrophotometer (Spectro UV-VIS Double Beam PC, Labomed, Los Angeles, CA, USA). Results are expressed as mg cyanidin-3-glucoside 100 g^−1^ of blackberry pomace extract [79].

#### 3.6.6. Volatile Organic Compounds of Extracts

The Heracles II e-nose (Alpha M.O.S., Toulouse, France), validated for ultrafast gas chromatography, was used to analyze the VOCs of the extracts of blackberry pomace according to the method described by Wojtasik-Kalinowska et al. [80]. Glass vials (20 mL) capped with a Teflon-faced silicone rubber cap were used for the sample preparation. The vials with 0.5 g of extract were placed in the automated sampler. Incubation of the vials was performed at 50 °C for 10 min with agitation at 500 rpm. The headspace sample of 2500 µL was injected (the injector temperature was 200 °C) into the GC using two columns with varying polarity. Non-polar MXT-5 (5% diphenyl) and semi-polar MXT-1701 (14% cyanopropylphenyl) 10 m columns with 0.18 mm internal diameter and two 270 °C flame ionization detectors (FID) were used. The injections were performed in 3 replicates. An alkane solution (*n*-butane to *n*-hexadecane) was used for calibration and conversion of the retention times to Kovat’s indices and identification of the volatile compounds using the AroChemBase database.

### 3.7. Statistical Analysis

Extraction experiments and phytochemical composition analysis were performed in triplicate and data are expressed as mean ± standard deviation. Statistical analyses were performed using one-way and two-way analysis of variance (ANOVA). Significant differences (*p* < 0.05) between samples were assessed by Fisher’s post hoc test. Principal Component Analysis (PCA) was performed to determine the influence of cultivar and the extraction method on the aroma profile of blackberry pomace extracts using the Alpha M.O.S. Heracles II device.

## 4. Conclusions

Blackberry pomace is a processing waste consisting mainly of seeds and its lipophilic extracts show a balanced profile of n-6/n-3 fatty acids and high content of PUFA fatty acids and carotenoids. The yield of the extracts is independent of the cultivar and is significantly influenced by the method of extraction. Meanwhile, the qualitative composition of the extract depends not only on the method of extraction, but also highly on the cultivar of the blackberry. The lipid composition of the extracts presents a high content of polyunsaturated fatty acids; one of them, and probably the most important, is C18:2. The highest content of PUFAs regardless of the extraction method, was in ‘Orkan’, and MUFAs in ‘Brzezina’ cultivar blackberry extracts. The highest amounts of fatty acids, chlorophylls, and carotenoids were determined in the Soxhlet with *n*-hexane and SC-CO_2_ extracts; therefore, these two extracts are the most valuable in terms of the compounds we determined in this study, but the environmental impact of the solvent used should be evaluated. Cultivar and extraction methods have significant influence on the content of carotenoids and chlorophylls in the extracts. The extracts are therefore not only valued for their polyunsaturated fatty acid composition, but are rich in compounds with coloring properties that can add value when used in different industries. The highest amounts of fatty acids, carotenoids, and chlorophylls were determined in the ‘Orkan’ cultivar extracts. The extraction of lipophilic compounds from blackberry pomace is proposed as an option to obtain high quality extracts with high nutritional value, and low peroxide values. Therefore, due to its rich lipid composition, blackberry extract is a valuable food ingredient that enhances the functionality of food products, contributes to a sustainable food industry chain in accordance with the zero-waste concept, and can also be used in medicine or cosmetics. 

## Figures and Tables

**Table 1:** Extract yield of blackberry pomace from different cultivars using different extraction methods, %.

Extraction Method	Soxhlet with Ethanol	Soxhlet with *n*-Hexane	SC-CO_2_
Cultivar			
‘Orkan’	44.16 ± 4.21 a *	5.87 ± 0.23 b	5.04 ± 0.25 b
‘Polar’	41.88 ± 1.13 a	4.97 ± 0.03 b	4.09 ± 0.03 b
‘Brzezina’	42.66 ± 4.67 a	4.93 ± 0.08 b	4.30 ± 0.27 b

* Mean values ± standard deviation, letters in the rows and columns indicate significant differences when *p* < 0.05.

**Table 2:** Peroxide value of different cultivars of blackberry pomace extracts, mEqkg^−1^.

Extraction Method	Soxhlet with Ethanol	Soxhlet with *n*-Hexane	SC-CO_2_
Cultivar			
‘Orkan’	1.67 ± 0.18 ef *	3.76 ± 1.31 a	2.70 ± 0.25 bc
‘Polar’	1.76 ± 0.03 def	3.80 ± 0.10 a	2.43 ± 0.16 cde
‘Brzezina’	1.53 ± 0.14 f	3.39 ± 0.09 ab	2.51 ± 0.11 cd

* Mean values ± standard deviation, letters in the rows and columns indicate significant differences when *p* < 0.05.

**Table 3:** Antioxidant activity, total phenol content (TPC), total flavonoid content (TFC) and total anthocyanin content (TAC) in ethanol Soxhlet extracts of blackberry pomace.

Cultivar	‘Orkan’	‘Polar’	‘Brzezina’
TPC, mg 100 g^−1^	313.18 ± 6.01 b *	313.04 ± 16.95 b	347.17 ± 14.63 a
TFC, mg 100 g^−1^	155.23 ± 5.46 a	133.75 ± 3.58 b	158.89 ± 14.60 a
TAC, mg 100 g^−1^	97.06 ± 2.93 a	26.33 ± 0.53 c	69.89 ± 3.48 b
IC_50_, µg mL^−1^	119.76 ± 0.47 b	124.01 ± 0.21 a	105.74 ± 1.04 c

* Mean values ± standard deviation, letters in the rows indicate significant differences when *p* < 0.05.

**Table 4:** Percentage of fatty acid groups in blackberry pomace lipophilic extracts, % from total fatty acid content.

	‘Orkan’			‘Polar’			‘Brzezina’			*p* Value		
Fatty Acids/Extraction Method	Soxhlet with Ethanol	Soxhlet with *n*-Hexane	SC- CO_2_	Soxhlet with Ethanol	Soxhlet with *n*-Hexane	SC- CO_2_	Soxhlet with Ethanol	Soxhlet with *n*-Hexane	SC- CO_2_	Cultivar	Extraction Method	Cultivar × Extraction Method
SFA	21.64 ± 0.16 b *	10.20 ± 0.21 c	10.27 ± 0.33 c	20.88 ± 1.02 b	10.64 ± 0.07 c	10.36 ± 0.12 c	25.78 ± 7.01 a	10.01 ± 0.72 c	10.25 ± 0.40 c	0.4003	0.0001	0.2771
MUFA	15.47 ± 0.02 b	11.25 ± 0.61 b	11.56 ± 0.92 b	15.26 ± 0.04 b	16.67 ± 0.06 b	16.11 ± 0.34 b	26.76 ± 11.05 a	14.34 ± 1.58 b	14.02 ± 1.47 b	0.0185	0.0124	0.0314
PUFA	62.90 ± 0.18 d	78.55 ± 0.82 a	78.17 ± 1.25 a	63.87 ± 0.98 d	72.69 ± 0.13 c	73.54 ± 0.21 bc	47.46 ± 4.04 e	75.65 ± 2.30 abc	75.74 ± 1.86 ab	0.0001	0.0001	0.0001
Omega- 3 acids	13.93 ± 0.08 a	13.35 ± 0.18 a	13.16 ± 0.23 ab	11.43 ± 0.09 cd	10.81 ± 0.04 d	10.60 ± 0.20 d	10.61 ± 1.36 d	12.30 ± 0.61 bc	12.27 ± 0.61 bc	0.0001	0.7946	0.0024
Omega- 6 acids	46.43 ± 0.32 e	65.01 ± 0.62 a	64.81 ± 1.02 bc	50.83 ± 0.93 d	61.72 ± 0.09 c	62.80 ± 0.02 c	33.65 ±1.38 f	63.18 ± 1.70 bc	63.28 ± 1.24 bc	0.0001	0.0001	0.0001
Omega- 9 acids	17.27 ± 0.06 b	10.96 ± 0.58 c	11.29 ± 0.91 c	16.20 ± 0.01 bc	16.36 ± 0.04 bc	15.80 ± 0.35 bc	28.97 ± 9.75 a	14.02 ± 1.57 bc	13.73 ± 1.43 bc	0.0070	0.0002	0.0064
MUFA/ PUFA	0.25	0.14	0.15	0.24	0.23	0.22	0.56	0.19	0.19			
PUFA/ SFA	2.91	7.70	7.61	3.06	6.83	7.10	1.84	7.56	7.39			
n-6/n-3	3.33	4.87	4.92	4.45	5.71	5.92	3.17	5.14	5.16			
U/S	3.62	8.80	8.74	3.79	8.40	8.65	2.88	8.99	8.76			

SFA—saturated fatty acids; MUFA—monounsaturated fatty acids; PUFA—polyunsaturated fatty acids; U/S—unsaturated fatty acid and saturated fatty acid ratio. * Mean values ± standard deviation, letters in the rows indicate significant differences when *p* < 0.05.

**Table 5:** Carotenoid and chlorophyll content in different blackberry cultivars’ lipophilic extracts; mg 100 g^−1^ of extract.

	‘Orkan’			‘Polar’			‘Brzezina’			*p* Value		
Name	Soxhlet with Ethanol	Soxhlet with *n*-Hexane	SC- CO_2_	Soxhlet with Ethanol	Soxhlet with *n*-Hexane	SC- CO_2_	Soxhlet with Ethanol	Soxhlet with *n*-Hexane	SC- CO_2_	Cultivar	Extraction Method	Cultivar × Extraction Method
	Carotenoids											
Zeaxanthin	6.87 ± 0.23 f *	66.13 ± 2.68 b	43.59 ± 1.22 e	5.89 ± 0.05 f	88.14 ± 2.27 a	51.49 ± 1.51 c	5.56 ± 0.28 f	48.33 ± 0.79 d	49.24 ± 0.98 cd	0.0001	0.0001	0.0001
Lutein	5.81 ± 0.93 f	535.9 ± 7.17 a	15.91 ± 0.20 e	4.07 ± 0.21 f	266.18 ± 10.31 b	33.45 ± 1.83 d	2.17 ± 0.35 f	129.68 ± 1.61 c	21.72 ± 0.75 e	0.0001	0.0001	0.0001
β-cryptoxanthin	0.72 ± 0.00 f	6.14 ± 0.14 a	4.45 ± 0.05 d	0.36 ± 0.02 g	4.55 ± 0.05 d	3.88 ± 0.07 e	0.37 ± 0.00 g	5.39 ± 0.10 b	4.98 ± 0.08 c	0.0001	0.0001	0.0001
α-carotene	1.69 ± 0.02 f	52.57 ± 0.45 a	36.55 ± 1.29 d	2.71 ± 0.18 f	46.96 ± 3.05 b	22.22 ± 1.49 e	1.33 ± 0.04 f	41.05 ± 2.38 c	36.11 ± 0.39 d	0.0001	0.0001	0.0001
β-catotene	40.31 ± 1.96 d	170.83 ± 4.53 a	117.63 ± 6.16 b	20.51 ± 0.99 e	162.35 ± 8.96 a	73.32 ± 1.30 c	26.33 ± 9.32 de	161.79 ± 21.74 a	167.9 ± 8.06 a	0.0001	0.0001	0.0001
	Chlorophylls											
Chlorophyll *a*	0.57 ± 0.02 e	8.37 ± 0.19 a	4.98 ± 0.12 cd	0.79 ± 0.03 e	5.94 ± 0.30 b	4.71 ± 0.02 d	0.62 ± 0.01 e	5.78 ± 0.26 b	5.19 ± 0.11 c	0.0001	0.0001	0.0001
Chlorophyll *b*	0.84 ± 0.05 e	7.07 ± 0.37 b	4.79 ± 0.17 d	0.85 ± 0.03 e	7.45 ± 0.23 a	4.68 ± 0.32 d	0.73 ± 0.01 e	5.85 ± 0.23 c	4.86 ± 0.08 d	0.0001	0.0001	0.0001

* Mean values ± standard deviation, letters in the rows indicate significant differences when *p* < 0.05.

## Data Availability

Data are contained within the article.

## Supplementary Materials

The following supporting information can be downloaded at: https://www.mdpi.com/article/10.3390/plants13202931/s1: Table S1: Fatty acid composition of blackberry pomace lipophilic extracts, % from a total fatty acids content.
