# Supplementary material for: Supercritical CO2 and Conventional Extraction of Bioactive Compounds from Different Cultivars of Blackberry (Rubus fruticosus L.) Pomace
Source: Plants (Basel). 2024 Oct 19;13(20):2931. doi: 10.3390/plants13202931 (PMC11511262; doi:10.3390/plants13202931)
Supplement: Supplementary file 1 [file plants-13-02931-s001.zip › plants-3244025-supplementary.pdf]

Table S1. Fatty acid composition in blackberry pomace lipophilic extracts, % from a total fatty acids content

	**'Orkan'**			**'Polar'**			**'Brzezina'**			**p Value**		
C:D	Soxhlet with ethanol	Soxhlet with *n*-hexane	SC-CO_2	Soxhlet with ethanol	Soxhlet with *n*-hexane %	SC-CO_2	Soxhlet with ethanol	Soxhlet with *n*-hexane	SC-CO_2	Cultivar	Extraction method	Cultivar x extraction method
C4:0	0.00 ± 0.00 c*	0.14 ± 0.03 b	0.16 ± 0.01 b	0.15 ± 0.04 b	0.32 ± 0.01 a	0.21 ± 0.11 ab	0.24 ± 0.02 ab	0.24 ± 0.01 ab	0.29 ± 0.06 a	0.0006	0.0091	0.1091
C6:0	0.82 ± 0.33 bc	0.15 ± 0.04c	0.17 ± 0.01 c	1.76 ± 0.67 ab	0.22 ± 0.01 c	0.20 ± 0.06 c	2.25 ± 1.26 a	0.19 ± 0.00 c	0.22 ± 0.01 c	0.2270	0.0007	0.2938
C8:0	0.32 ± 0.04 b	0.39 ± 0.01 b	0.40 ± 0.02 b	0.84 ± 0.41 a	0.14 ± 0.02 b	0.29 ± 0.01 b	0.95 ± 0.23 a	0.11 ± 0.01 b	0.20 ± 0.02 b	0.8054	0.0009	0.0138
C10:0	0.00 ± 0.00 e	0.53 ± 0.02 a	0.54 ± 0.04 a	0.05 ± 0.07 e	0.20 ± 0.03 cd	0.39 ± 0.02 b	0.00 ± 0.00 e	0.15 ± 0.01 d	0.27 ± 0.02 c	0.0001	0.0001	0.0001
C11:0	0.44 ± 0.04 ab	0.08 ± 0.01 c	0.09 ± 0.01 c	0.40 ± 0.04 abc	0.23 ± 0.00 bc	0.13 ± 0.04 bc	0.71 ± 0.43 a	0.21 ± 0.01 bc	0.19 ± 0.01 bc	0.1906	0.0026	0.5999
C12:0	1.91 ± 0.14 b	0.07 ± 0.01 c	0.08 ± 0.01 c	2.79 ± 0.19 ab	0.09 ± 0.00 c	0.08 ± 0.01 c	3.86 ± 2.08 a	0.11 ± 0.02 c	0.10 ± 0.01 c	0.2965	0.0001	0.3522
C13:0	0.21 ± 0.06 e	1.41 ± 0.03 a	1.42 ± 0.01 a	0.71 ± 0.35 d	1.01 ± 0.01 bcd	1.28 ± 0.06 ab	0.81 ± 0.21 cd	1.04 ± 0.03 bc	1.14 ± 0.04 ab	0.9719	0.0001	0.0038
C14:0	0.30 ± 0.02 a	0.07 ± 0.00 b	0.07 ± 0.01 b	0.21 ± 0.01 a	0.07 ± 0.00 b	0.06 ± 0.00 b	0.29 ± 0.16 a	0.08 ± 0.01 b	0.08 ± 0.01 b	0.4915	0.0001	0.7621
C14:1 cis-9	0.62 ± 0.08 b	0.26 ± 0.02 b	0.27 ± 0.01 b	1.87 ± 0.23 a	0.18 ± 0.00 b	0.24 ± 0.02 b	3.02 ± 1.55 a	0.19 ± 0.00 b	0.22 ± 0.01 b	0.0897	0.0005	0.0479
C15:0	5.32 ± 0.13 a	0.02 ± 0.00 c	0.02 ± 0.00 c	2.86 ± 0.64 b	0.02 ± 0.00 c	0.2 ± 0.00 c	5.25 ± 2.79 a	0.02 ± 0.00 c	0.02 ± 0.00 c	0.2737	0.0001	0.2806
C15:1 cis-10	0.00 ± 0.00 b	0.00 ± 0.00 b	0.00 ± 0.00 b	0.09 ± 0.01 a	0.01 ± 0.01 b	0.00 ± 0.00 b	0.00 ± 0.00 b	0.00 ± 0.00 b	0.00 ± 0.00 b	0.0001	0.0001	0.0001
C16:0	7.46 ± 0.09 a	3.68 ± 0.08 c	3.66 ± 0.16 c	6.00 ± 0.04 b	4.02 ± 0.03 c	3.88 ± 0.01 c	6.52 ± 1.65 ab	3.92 ± 0.27 c	3.82 ± 0.08 c	0.6691	0.0001	0.2422
C16:1 cis-9	0.16 ±	0.06 ±	0.05 ±	0.18 ±	0.06 ±	0.06 ±	0.18 ±	0.05 ±	0.05 ±	0.8513	0.0001	0.9378

	0.01 a	0.01 b	0.00 b	0.01 a	0.01 b	0.01 b	0.07 a	0.01 b	0.01 b			
C17:0	0.14 ± 0.01 a	0.07 ± 0.00 bc	0.07 ± 0.00 bc	0.12 ± 0.01 ab	0.08 ± 0.00 bc	0.08 ± 0.00 bc	0.05 ± 0.07 c	0.08 ± 0.01 bc	0.08 ± 0.01 bc	0.1709	0.1709	0.1243
C17:1 cis-10	0.30 ± 0.01 a	0.05 ± 0.00 b	0.05 ± 0.00 b	0.18 ± 0.01 ab	0.06 ± 0.00 b	0.05 ± 0.00 b	0.37 ± 0.26 a	0.06 ± 0.01 b	0.05 ± 0.00 b	0.4948	0.0019	0.5316
C18:0	2.35 ± 0.04 a	2.03 ± 0.11 a	2.07 ± 0.18 a	2.11 ± 0.04 a	2.35 ± 0.01 a	2.36 ± 0.05 a	2.09 ± 0.23 a	2.15 ± 0.30 a	2.18 ± 0.16 a	0.3192	0.9576	0.1628
C18:1	13.55 ± 0.20 a	10.41 ± 0.85 a	10.70 ± 1.30 a	12.61 ± 0.45 a	15.93 ± 0.04 a	15.34 ± 0.47 a	22.24 ± 17.52 a	13.61 ± 2.21 a	13.20 ± 2.02 a	0.4055	0.6286	0.6319
C18:2	46.43 ± 0.45 c	64.99 ± 0.88 a	64.77 ± 1.44 a	50.62 ± 1.36 b	63.16 ± 0.12 a	62.73 ± 0.04 a	33.33 ± 1.72 d	63.11 ± 2.38 a	63.21 ± 1.73 a	0.0001	0.0001	0.0001
C19:0	0.44 ± 0.04 a	0.04 ± 0.02 c	0.04 ± 0.00 c	0.23 ± 0.06 b	0.09 ± 0.01 c	0.05 ± 0.00 c	0.23 ± 0.13 b	0.07 ± 0.01 c	0.06 ± 0.01 c	0.1672	0.0001	0.0186
C18:3	13.16 ± 0.00 a	13.22 ± 0.24 a	13.05 ± 0.32 a	10.77 ± 0.16 bc	10.65 ± 0.06 c	10.52 ± 0.29 c	9.67 ± 1.39 c	12.14 ± 0.92 ab	12.16 ± 0.87 ab	0.0002	0.1186	0.0530
C20:0	0.66 ± 0.03 b	1.19 ± 0.02 a	1.18 ± 0.06 a	0.43 ± 0.02 b	1.31 ± 0.02 a	1.07 ± 0.01 a	0.61 ± 0.01 b	1.19 ± 0.23 a	1.23 ± 0.08 a	0.4805	0.00001	0.2960
C20:1	0.28 ± 0.03 a	0.38 ± 0.01 a	0.38 ± 0.01 a	0.23 ± 0.01 a	0.36 ± 0.01 a	0.34 ± 0.00 a	0.45 ± 0.33 a	0.40 ± 0.01 a	0.38 ± 0.01 a	0.3198	0.6617	0.7096
C20:2	0.09 ± 0.01 a	0.12 ± 0.03 a	0.14 ± 0.01 a	0.05 ± 0.07 a	0.09 ± 0.00 a	0.08 ± 0.01 a	0.60 ± 0.84 a	0.11 ± 0.01 a	0.11 ± 0.03 a	0.4685	0.6361	0.5762
C21:0	0.29 ± 0.03 a	0.04 ± 0.01 b	0.03 ± 0.00 b	0.04 ± 0.05 b	0.04 ± 0.00 b	0.03 ± 0.01 b	0.00 ± 0.00 b	0.04 ± 0.01 b	0.03 ± 0.00 b	0.0001	0.0001	0.0001
C20:3 8,11,14	0.24 ± 0.04 b	0.02 ± 0.00 b	0.01 ± 0.00 b	0.74 ± 0.37 a	0.03 ± 0.00 b	0.02 ± 0.00 b	0.85 ± 0.25 a	0.03 ± 0.01 b	0.02 ± 0.00 b	0.0785	0.0001	0.0713
C20:4	0.00 ± 0.00 c	0.03 ± 0.01 b	0.04 ± 0.01 a	0.00 ± 0.00 c	0.04 ± 0.00 a	0.04 ± 0.00 a	0.00 ± 0.00 c	0.04 ± 0.01 a	0.04 ± 0.00 a	0.0480	0.0001	0.2066

C20:3 11,14,17	2.22 ± 0.06 a	0.05 ± 0.00 c	0.06 ± 0.01 c	0.83 ± 0.23 b	0.05 ± 0.00 c	0.04 ± 0.00 c	1.78 ± 0.76 a	0.04 ± 0.01 c	0.07 ± 0.01 c	0.0364	0.0001	0.0248
C22:0	0.76 ± 0.07 a	0.25 ± 0.01 c	0.24 ± 0.01 c	0.34 ± 0.04 c	0.41 ± 0.00 bc	0.21 ± 0.02 c	0.66 ± 0.29 ab	0.35 ± 0.15 c	0.29 ± 0.04 c	0.2332	0.0014	0.0542
C22:1	0.46 ± 0.05 a	0.04 ± 0.02 bcd	0.05 ± 0.01 bc	0.00 ± 0.00 d	0.03 ± 0.01 bcd	0.03 ± 0.00 bcd	0.00 ± 0.00 d	0.01 ± 0.01 cd	0.06 ± 0.02 b	0.0001	0.0001	0.0001
C20:5	0.29 ± 0.04 ab	0.02 ± 0.00 c	0.02 ± 0.00 c	0.18 ± 0.01 b	0.00 ± 0.00 c	0.01 ± 0.01 c	0.34 ± 0.19 a	0.00 ± 0.00 c	0.00 ± 0.00 c	0.3701	0.0001	0.4623
C22:2	0.00 ± 0.00 c	0.00 ± 0.00 c	0.01 ± 0.01 c	0.21 ± 0.04 ab	0.02 ± 0.00 c	0.02 ± 0.01 c	0.33 ± 0.23 a	0.04 ± 0.03 bc	0.03 ± 0.01 bc	0.0583	0.0109	0.1321
C23:0	0.24 ± 0.07 b	0.03 ± 0.00 b	0.03 ± 0.00b	0.89 ± 0.42 a	0.04 ± 0.00 b	0.03 ± 0.00 b	1.02 ± 0.29 a	0.04 ± 0.01 b	0.17 ± 0.01 b	0.0451	0.0001	0.0725
C24:0	0.00 ± 0.00 b	0.04 ± 0.00 b	0.04 ± 0.00 b	0.30 ± 0.00 a	0.03 ± 0.00 b	0.03 ± 0.01 b	0.28 ± 0.21 a	0.06 ± 0.01 b	0.06 ± 0.00 b	0.0541	0.0058	0.0400
C24:1	0.11 ± 0.07 b	0.07 ± 0.00 b	0.08 ± 0.01 b	0.11 ± 0.15 b	0.06 ± 0.01 b	0.06 ± 0.01 b	0.51 ± 0.34 a	0.04 ± 0.00 b	0.07 ± 0.00 b	0.1786	0.0529	0.1282
C22:6	0.49 ± 0.08 a	0.11 ± 0.01 b	0.10 ± 0.01 b	0.48 ± 0.02 a	0.16 ± 0.00 b	0.08 ± 0.00 bc	0.60 ± 0.34 a	0.16 ± 0.06 b	0.12 ± 0.01 b	0.6496	0.0002	0.9352

*Mean values ± standard deviation, letters in the rows indicates significant differences when $p < 0.05$
